# Psychosis following traumatic brain injury: A case study and a brief overview

**DOI:** 10.1192/j.eurpsy.2024.603

**Published:** 2024-08-27

**Authors:** H. Khiari, A. Hakiri, S. Rouai, R. Ghachem

**Affiliations:** Psychiatry B, Razi hospital, Mannouba, Tunisia

## Abstract

**Introduction:**

Psychosis resulting from traumatic brain injury (TBI) is a relatively uncommon but potentially severe and disabling outcome. The complex relationship between TBI and the onset of psychosis is marked by significant scientific uncertainty and differing opinions.

**Objectives:**

To investigate the occurrence of psychosis following traumatic brain injury (TBI) and explore the intricate relationship between TBI and the development of psychosis.

**Methods:**

A comprehensive case report was conducted on a 38-year-old patient who, after a severe TBI at the age of 23, exhibited signs of psychosis. Developmental history, family background, clinical assessments, magnetic resonance imaging (MRI), and electroencephalogram (EEG) results were analyzed.

**Results:**

The patient, at the time of writing aged 38, was born at full term with a regular presentation and uneventful delivery, with no indications of perinatal or obstetric complications. Developmentally, he reached all milestones within the expected range, and there were no significant premorbid characteristics. There was no family history of schizophrenia in a first- or second-degree relative; a paternal cousin had had psychosis-like symptoms, but reportedly remained well without any medication.

At the age of 23, the patient was knocked from his motorcycle by a car and sustained a severe traumatic brain injury (TBI), with initial loss of consciousness and was in a coma state for approximatively a month, with later sequelae of cerebellar syndrome and predominant right-sided pyramidal syndrome.

Magnetic resonance imaging (MRI) a year following the TBI showed sequelae of bifrontal and temporal contusion lesions.

An EEG did not indicate any evidence of epilepsy, and a repeat EEG 14 years later revealed no diagnostic abnormality.

A year after the accident, his surroundings have noticed social withdrawal, a turning inward with a religious fervor, and persecutory remarks focused on his brother. At the age of 26, he presented to a psychiatric service having auditory hallucinations. He was deluded, believing himself to be a prophesied redeemer figure who is expected to appear and bring justice and righteousness to the world. He had an inappropriate affect. A diagnosis of schizophrenia was made, and neuroleptics prescribed. His auditory hallucinations faded, but the subsequent course was of repeated episodes of florid psychosis requiring maintenance neuroleptic treatment, eventually haloperidol decanoate (150mg monthly).

**Conclusions:**

Psychosis following TBI is an uncommon yet potentially severe consequence, carrying the risk of significant debilitation. The relationship between TBI and psychosis is complex, but notable distinctions exist in clinical, epidemiological, and neurobiological aspects when compared to primary psychotic disorders.

**Disclosure of Interest:**

None Declared

